# Does Postevacuation **β**-Human Chorionic Gonadotropin Level Predict the Persistent Gestational Trophoblastic Neoplasia?

**DOI:** 10.1155/2014/494695

**Published:** 2014-03-24

**Authors:** Azam Sadat Mousavi, Samieh Karimi, Mitra Modarres Gilani, Setareh Akhavan, Elahe Rezayof

**Affiliations:** ^1^Gynecology Oncology, Tehran University of Medical Sciences (TUMS), Tehran, Iran; ^2^Hormozgan Fertility & Infertility Research Center, Hormozgan University of Medical Sciences, Bandar Abbas 7914964157, Iran; ^3^Vali-e-Asr Reproductive Health Research Center, Tehran University of Medical Sciences (TUMS), Tehran, Iran

## Abstract

**β**-human chorionic gonadotropin (HCG) level is not a reliable marker for early identification of persistent gestational trophoblastic neoplasia (GTN) after evacuation of hydatidiform mole. Thus, this study was conducted to evaluate **β**-HCG regression after evacuation as a predictive factor of malignant GTN in complete molar pregnancy. *Methods*. In this cross-sectional study, we evaluated a total of 260 patients with complete molar pregnancy. Sixteen of the 260 patients were excluded. Serum levels of HCG were measured in all patients before treatment and after evacuation. HCG level was measured weekly until it reached a level lower than 5 mIU/mL. *Results*. The only predictors of persistent GTN are HCG levels one and two weeks after evacuation. The cut-off point for the preevacuation HCG level was 6000 mIU/mL (area under the curve, AUC, 0.58; sensitivity, 38.53%; specificity, 77.4%), whereas cut-off points for HCG levels one and two weeks after evacuation were 6288 mIU/mL (AUC, 0.63; sensitivity, 50.46%; specificity, 77.0%) and 801 mIU/mL (AUC, 0.80; sensitivity, 79.82%; specificity, 71.64%), respectively. *Conclusion*. The rate of decrease of HCG level at two weeks after surgical evacuation is the most reliable and strongest predictive factor for the progression of molar pregnancies to persistent GTN.

## 1. Introduction

Persistent gestational trophoblastic neoplasia (GTN) includes hydatidiform mole, invasive mole, choriocarcinoma, and placental site tumor derived from the placenta; persistent GTN is a curable disease but can develop into a life-threatening malignancy [1–3]. Dilation-curettage and chemotherapy are suitable treatments for low-risk GTN [4, 5].

Postmolar GTN is defined by clinical and laboratory criteria. Persistent GTN specifically refers to GTNs with potential for tissue invasion and metastasis.

Human chorionic gonadotropin (HCG) is a glycoprotein hormone comprising two subunits, alpha and beta, and is an important index for pregnancy and gestational trophoblastic disease [6, 7].

Serial evaluation of HCG can be used for diagnosis of normal and abnormal pregnancies [6, 8–11].

A comprehensive study was performed to identify predictive factors in progressive normal pregnancy, molar pregnancy, and invasive mole to malignant disease. The risk factors that cannot strongly predict persistent GTN include the following: age, age of pregnancy, positive past medical history of molar pregnancy, HCG titer of more than 100,000, and theca lutein cyst > 6 cm [12–14].

A reliable marker for early identification of persistent GTN after evacuation of hydatidiform mole is not available. Thus, this study was performed to evaluate *β*-HCG regression after evacuation as a predictive factor of malignant GTN in complete molar pregnancy.

## 2. Method and Material

Patients with complete molar pregnancies were included in the study, and patients who received chemoprophylaxis were excluded. Patients who showed noncompliance to the follow-up process were considered dropped cases and were excluded. In this cross-sectional study, a total of 260 patients with complete molar pregnancy who were referred to the Gynecology Oncology Department of the Imam Khomeini Hospital of Tehran University of Medical Sciences (Iran) were evaluated. Sixteen of the 260 patients were excluded. Seven patients who received prophylactic chemotherapy before molar evacuation and nine patients who discontinued the follow-up process were also excluded.

Information on age, gestational age, gravidity, parity, and history of prophylactic chemotherapy before molar evacuation was obtained from the patients. Serum level of HCG was measured in all patients before treatment and after evacuation. HCG was measured weekly until it reached a level lower than 5 mIU/mL. Spontaneous remission was diagnosed in patients who had undetectable HCG for three weeks after evacuation.

Persistent GTN was defined based on the following 2000 FIGO criteria.

1. Less than 10% decrease in serum HCG level for at least four values for longer than three weeks.
2. Higher than 10% increase of serum HCG level for three values for longer than two consecutive weeks (e.g., days 1, 7, and 14).
3. Persistence of detectable serum HCG for more than six months after molar evacuation.

Patients were divided into two groups: group A (remission) and group B (persistent GTN). Analysis was done with SPSS 20.0 software. Descriptive statistics, Chi-Square, and Independent Samples* t*-test were used for comparing two groups. Also ROC curve was used for calculation of cut-off point for sensitivity and specificity of the tests.

## 3. Results

A total of 109 patients (44.7%) were diagnosed with persistent GTN. The mean age of the patients was 27.28 ± 8.1 years old (in the range 15 years old to 54 years old). The mean ages of the patients in groups A and B were 27.01 ± 8.3 and 27.61 ± 7.9 years old, respectively. Age of individuals in the two groups did not significantly differ (*P* < 0.05).

In Table 1, age, parity, gestational age, preevacuation HCG level, and HCG levels after one and two weeks of patients diagnosed with persistent GTN and those of undiagnosed patients were compared.

The median HCG levels before evacuation and one and two weeks after evacuation in groups A and B were 89,595 and 29,000, 1893 and 6300, and 427 and 2,090 mIU/mL, respectively.

As shown in Table 1, the only predictors of persistent GTN were HCG levels at one and two weeks after evacuation. Other factors were not related to persistent GTN.

**Figure 1:**
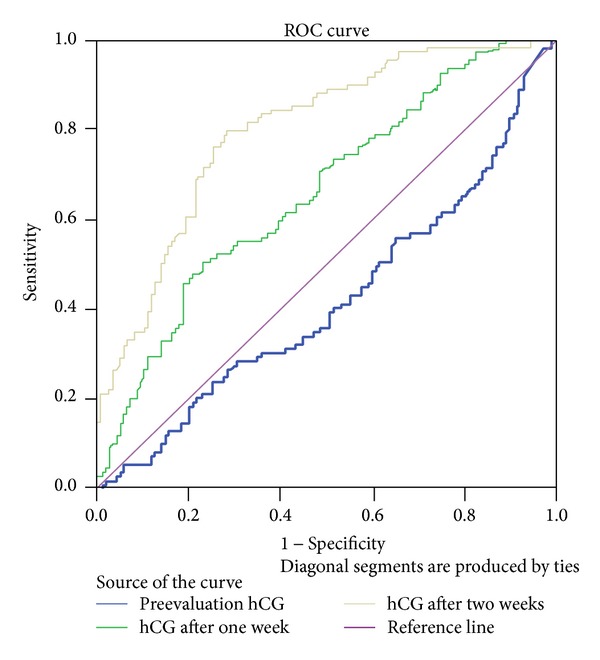
Diagnostic value of preevacuation levels of HCG and levels after one and two weeks in patients with persistent GTN.

Figure 1 shows the receiving operator characteristic curve for diagnostic value of preevacuation levels of HCG and levels after one and two weeks in patients with persistent GTN.

The area under the curve (AUC) and related data are shown in Table 2. According to this table, the cut-off point for the preevacuation HCG level was 6,000 mIU/mL (AUC, 0.58; sensitivity, 38.53%; specificity, 77.4%). The HCG level at one week after evacuation was 6,288 mIU/mL (AUC, 0.63; sensitivity, 50.46%; specificity, 77.0%), and HCG level at two weeks after evacuation was 801 mIU/mL (AUC, 0.80; sensitivity, 79.82%; specificity, 71.64%).

The cut-off point for ratio of preevacuation HCG level to HCG level after one week was 13.33 (AUC, 0.73; sensitivity, 69.72%; specificity, 68.89%) and that for the ratio of preevacuation HCG level to HCG after two weeks was 250 (AUC, 0.78; sensitivity, 88.99%; specificity, 56.72%).

The cut-off point for the ratio of the HCG level one week after evacuation to that two weeks after evacuation was 4.16 (AUC, 0.70; sensitivity, 69.72%; specificity, 71.64%), as shown in Table 2. The predictive values of HCG level in persistent GTN prediction is summarized in Table 3.

## 4. Discussion

To find a marker for the prediction of postmolar persistent GTN, we studied the serum HCG levels of 260 patients with molar pregnancy. The goal of this study was to asses HCG level in patients with complete molar pregnancy to predict the occurrence of persistent GTN after surgical evacuation of molar pregnancies.

A total of 260 patients were evaluated, among which 44.7% were diagnosed with persistent GTN. In comparison, this rate in studies by Kang et al. [1] and Wolfberg et al. [12] was 24.2% and 15%, respectively.

Preevacuation HCG levels in the patients without GTN were higher than HCG levels in patients with persistent GTN. The significantly higher HCG levels in patient without GTN might be due to high gestational age and delay in diagnosis.

Significant relationships exist between persistent GTN and HCG levels one and two weeks after evacuation. This finding indicates that the most reliable predictor of GTN is the serial HCG level at one and two weeks after evacuation.

In Kang et al. [1], the cut-off points for the HCG level at one and two weeks after evacuation were 6,400 and 2,400 mIU/mL, respectively, and the cut-off point for the ratio of preevacuation HCG level to HCG level at two weeks after evacuation was 30. In the current study, the cut-off point for the preevacuation HCG level was 6,000 mIU/mL, and cut-off points of the HCG levels at one and two weeks after evacuation were 6,288 and 801 mIU/mL, respectively. The cut-off point for ratio of preevacuation HCG level to HCG level at two weeks after evacuation was 250.

In a study by Wolfberg et al. [12], the risk of progression to persistent GTN in women whose HCG level decreased to undetectable level was 0.2%. In our study, women whose HCG level decreased more than two weeks after evacuation showed progression to persistent GTN.

Khoo et al. [5] and Ayhan et al. [13] did not observe a significant and strong relationship between serum HCG level and risk of persistent GTN in molar pregnancies, but other papers showed the opposite. A significant relationship was found between serum HCG level and risk of GTN, and thus, serum HCG level may be useful for the early prediction of postmolar persistent GTN [8, 10, 15–17].

According to the results of this study, preevacuation HCG level did not have a significant relationship with progression to persistent GTN, but the observed HCG serum levels at one and two weeks after evacuation of molar pregnancy can be satisfactory predictors of GTN.

The rate of decrease of HCG level at two weeks after surgical evacuation is the most reliable and the strongest predictive factor for the progression of molar pregnancies to persistent GTN. The ratio of preevacuation HCG level to HCG level at two weeks after evacuation was a better predictive factor than the others (Table 2), and this ratio in the current study was 250. Results would allow physicians to assess the early prediction of persistent GTN in molar pregnancy by the determination of serial HCG level, particularly at two weeks after molar evacuation. Future prospective studies should focus on early prediction of persistent GTN.

## Figures and Tables

**Table 1:** Comparison of baseline characteristics of the patients, and HCG levels at preevacuation and after one and two weeks in the two groups.

	Persistent GTN (*n* = 109)	Remission group (*n* = 151)	*P* value
Age			
<40	98 (89.9%)	121 (89.6%)	0.558
≧40	11 (10.1%)	14 (10.4%)	
Parity			
Nulliparous	24 (22.9%)	34 (25.2%)	0.683
1	24 (22.9%)	34 (25.2%)	
≧2	24 (22.9%)	34 (25.2%)	
Gestational age	8.6 ± 1.3	6.2 ± 1.1	0.030
Preevacuation hCG	17,1420 ± 299,843	23,5764 ± 41,8102	0.178
hCG after one week	18,408 ± 36,311	7,966 ± 17,571	0.004
hCG after two weeks	15,811 ± 71,830	1,140 ± 2,041	0.019

**Table 2:** Cut-off point for HCG level in the prediction of malignant GTN.

	Cut of point	AUC (±SD)
Preevacuation (mIU/mL)	6,000	0.58 ± 0.04
One week after evacuation (mIU/mL)	6,288	0.63 ± 0.04
Two weeks after evacuation (mIU/mL)	801	0.80 ± 0.03
Ratio of preevacuation level to the level after one week	13.33	0.73 ± 0.03
Ratio of preevacuation HCG level to HCG level after two weeks	250	0.78 ± 0.03
Ratio of HCG level after one week to that after two weeks	4.16	0.70 ± 0.04

AUC: area under the curve.

**Table 3:** Predictive values of HCG level in persistent GTN prediction.

	Sensitivity	Specificity	PPV	NPV
Preevacuation (mIU/mL)	38.53	77.4	39.2	42.5
One week after evacuation (mIU/mL)	50.46	77	64	65
Two weeks after evacuation (mIU/mL)	79.82	71.64	69.6	81.4

PPV: positive predictive value, NPV: negative predictive value.
